# Study of Adhesion–Cohesive Interactions of Modified Bitumen Compositions

**DOI:** 10.3390/polym17020199

**Published:** 2025-01-14

**Authors:** Antonina Dyuryagina, Alyona Degert, Yuliya Byzova, Kirill Ostrovnoy, Alexandr Demyanenko, Aida Lutsenko, Tatyana Shirina

**Affiliations:** Department of Chemistry and Chemical Technology, Manash Kozybayev North Kazakhstan University, Petropavlovsk 150000, Kazakhstan; adyuryagina@inbox.ru (A.D.); helena.dgrt@bk.ru (A.D.); kostrovnoy@mail.ru (K.O.); demianenkoav@mail.ru (A.D.); l-a.13@mail.ru (A.L.); tshirina@internet.ru (T.S.)

**Keywords:** polymeric composites, bitumen modification, adhesion, cohesion, mathematical modeling, surfactant, polymer

## Abstract

The aim of the work was to study the effect of additive concentration on changes in the adhesive and cohesive strength of bitumen. To evaluate the effectiveness of modifiers in the composition of binary and triple bitumen systems in relation to mineral fillers of two grades, the method of determination of the adhesive efficiency and thermodynamic calculations of adhesion and cohesion work were used. The following compounds were used as additives: ***AS-2*** synthesized from the oil refining waste and ***AG-4I*** (waste sealing liquid). Adhesion–cohesion processes in modified bitumen systems are limited by the wetting effect of crushed stone and the intensity of intermolecular forces in the condensed phase of the binder. In the binary compositions, the addition of modifiers into bitumen significantly improves the cohesive strength and adhesive efficiency in relation to crushed stone. The introduction of ***AS-2*** into bitumen compositions with ***AG-4I*** increases adhesion efficiency and thermodynamic work of adhesion in relation to the filler surface. The adhesion efficiency and thermodynamic work of adhesion in the “bitumen-***AG-4I***-***AS-2***” system reach their maximum at ***C_AG-4I_*** = 3.0 g/dm^3^ and ***C_AS-2_*** = 1.5 g/dm^3^. In these concentration modes, the composition ensures maximum increase in adhesion efficiency (***A_KS_*** = 65.18%; ***A_KA_*** = 48.11%) and the greatest increase in thermodynamic work of adhesion (***W_A(KS)_*** = 15.79 mN/m; ***W_A(KA)_*** = 14.13 mN/m).

## 1. Introduction

The current level of road operation is associated with a continuous increase in the share of freight traffic and the intensity of vehicle traffic, which leads to an increase in dynamic loads on the asphalt–concrete road surface. Poor road bitumen quality is one of the primary causes of premature road surface degradation, which leads to a decrease in the cohesive strength and stability of the roadway, trackage, and other defects (cracks, subsidence, etc.) [1,2]. The penetration of dust and water particles into the cracks formed, combined with simultaneous temperature changes, accelerates this process of integrity disruption and eventually leads to the complete destruction of the pavement [3,4,5]. This is due to the fact that unmodified bitumen binders have a higher molecular weight and lower polarity than water, so they form a weak bond with surface of mineral filler and easily peel off in the presence of moisture [5,6,7,8].

To ensure the durability and achieve the high physicochemical characteristics of asphalt concrete, it is essential to explore novel scientific and technological approaches in the field of bitumen modification with various additives [1,3]. Surfactants, particularly cationic types, are among the most recognized and commonly used modifiers for improving adhesive properties in road construction—alkyldiamine [9], amidoamine, diamidoamine [10], heterocyclic amine [11], and octadecylamine [12]. To increase the cohesive strength, as numerous studies have shown, it is preferable to introduce polymer additives into the bitumen binder [1,13,14,15,16,17,18]. The polymer not only fills the space between bitumen asphaltenes, but it also creates a structural grid that increases the strength of intermolecular interactions inside bitumen, i.e., its strength [9,15,16]. As a result of the combined introduction of the polymer and surfactant into bitumen, a new structure is created, which leads to a change in the initial properties of the binder, and, as a result, the polarity of the composition changes. The polarity of the composition, in turn, will have a dominant effect on the cohesive interaction in the binder volume, as well as on the surface tension at the interfacial boundaries of bitumen with air and mineral filler. Thus, the introduction of modifying additives creates the possibility of fine regulation of adhesive–cohesive interactions in a multicomponent bitumen composition.

The criterion for assessing the effect of modification is the level of change in the adhesive–cohesive interactions of the bitumen binder. Depending on the strength of the adhesive bonds, adhesive detachment may occur (the entire coating breaks off), cohesive detachment (the coating or substrate breaks), mixed adhesive–cohesive detachment (the coating partially peels off and the adhesive or the substrate partially collapses) [19].

Indirect methods for determining adhesion have a long history of application and are the most numerous [5]. Those methods are based on two types of intermolecular interactions: intraphase (cohesion) and interphase (adhesion). The Dupré–Young Thermodynamic Equation (1) makes it possible to calculate the adhesion function (***W_A_***) [20] based on the results of experimental determination of the surface tension of a bitumen binder at the interface with air (***σ_l-g_***) and the equilibrium contact angle of wetting the surface of a mineral filler with bitumen (*θ*):

***W_A_* = *σ_l-g_*(1 + *cosθ*)** (1)

A quantitative characteristic of the cohesive interaction inside bitumen is the work of cohesion (***W_K_***), which is equal to twice the surface tension (2) [21]:

***W_K_* = 2*σ_l-g_*** (2)

From the analysis of the presented Dupré–Young Equation (1), it follows that the choice of surfactants to enhance the adhesion of bitumen to mineral surfaces should be based on the fact that they are mainly concentrated on the interfacial surface “bitumen–mineral filler” and not on the boundary of bitumen and air. It is with such a quantitative distribution of surfactants at the interfacial boundaries of a dispersed bitumen system that wetting will improve (***cosθ***→1) without significant changes in ***σ_l-g_***. Surfactants, concentrating on the surface of mineral filler, form an adsorption layer in polar groups facing the surface of the crushed stone, and hydrocarbon radicals form into the volume of bitumen, smoothing out the difference in polarities between the two phases. As a result, the contact angle of the wetting of the solid surface decreases, and the work of the adhesion increases. The additional introduction of a polymer additive should contribute to an increase in ***σ_l-g_***, which, in accordance with the Dupré–Young Equation (1), also entails an increase in the ***W_A_***—the work required to rupture the unit area of the interfacial surface layer.

In addition to thermodynamic determination of the ratio of equilibrium adhesion (***W_A_***) and equilibrium cohesion (***W_K_***), there are methods for determining the strength of coatings. These include methods of peeling, tearing off, or destroying the coating [22,23]. The adhesive strength of the bitumen coating, i.e., the adhesion of the binder to the surface of the mineral filler upon their contact, is characterized by the work of separation *A*_0_ or separation force *F_Q_* necessary to break the bonds between the adhesive and the substrate. When the coating is torn off or destroyed, the integrity of the coating itself, i.e., its cohesion, may be disrupted [24]. The separation of the coating can be carried out by simultaneous separation of the adhesive and the substrate over the entire contact area by the normal separation force or by sequential separation (peeling) by the normal force or tangential separation force. In these cases, the coating and the substrate are separated by such distances that exclude their further interaction. The adhesive strength can also be determined by shifting the coating relative to the substrate. The simultaneous separation method makes it possible to bring the adhesive strength as close as possible to equilibrium adhesion; however, it is applicable only with a small contact area, since it is difficult to create equal stresses over the entire cross-section of the sample [24]. The peeling method is the simplest, but the difference between adhesive strength and adhesion in this case is the greatest.

Methods for assessing the adhesion of bitumen binders must meet certain requirements: theoretical validity, high accuracy, and reproducibility of the results, ensuring the required sensitivity of the method by selecting the temperature and test time, the possibility of comparative evaluation of the original and modified bitumen, simplicity, and short duration in time. The lack of a single generally accepted method for assessing adhesion is currently explained by the fact that none of the numerous developed methods has specified above criteria, while the market for road building materials is constantly being updated with new types of adhesive additives, and a comparative analysis of their effects is difficult due to the imperfection of adhesion assessment methods. At the same time, the high adhesion values obtained in one of the methods have often not been confirmed by the results of other adhesion assessment methods [5].

The necessity to improve the adhesive and cohesive properties of binders in these areas stimulated our research on the creation of bitumen compositions using the cationic surfactant AC-2 [25] based on petrochemical by-products (KOH-92) and spent polymer materials requiring recycling (***AG-4I***).

The purpose of this work is to study the effect of modifier concentrations on intraphase and interphase changes in bitumen compositions based on a comparative analysis of two methods for their determination.

To accomplish this objective, the following tasks were established:

1. Investigation of the adhesive properties of the binary “bitumen-***AG-4I***”, “bitumen-***AS-2***” compositions, and the triple composition “bitumen-***AS-2***-***AG-4I***” according to the mass of the detached binder from the surface of two types of crushed stone;
2. Study of the effect of the concentration of modifiers on the change in the specific surface energy of “bitumen–air” and the wetting angle of mineral fillers;
3. Physicochemical determination of the adhesive and cohesive strength of binary and triple bitumen compositions from the concentration of additives;
4. Modeling of the effect of concentrations of ***AG-4I*** and ***AS-2*** on the thermodynamic work of adhesion and the adhesive efficiency of triple bitumen compositions.

## 2. Materials and Methods

### 2.1. Materials

When conducting research, we used the following:

1. Oxidized bitumen with a penetration grade of 100/130 produced by “Gazpromneft-Bitumen Kazakhstan” LLP, Shymkent, Kazakhstan. The composition and structural properties of the samples were analyzed using infrared (IR) spectroscopy techniques.

**Figure 1:**
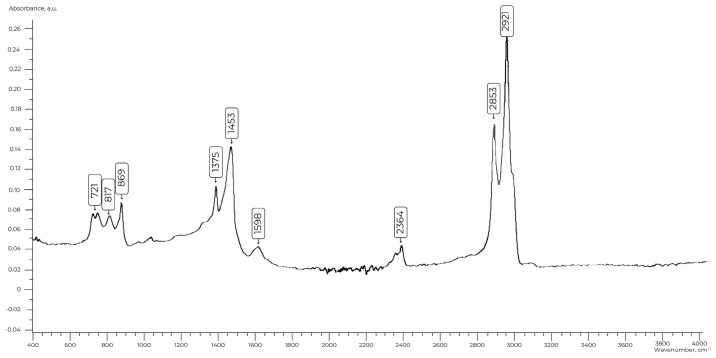
IR spectrum of bitumen.

In the IR spectra of the bitumen (Figure 1), a band corresponding to the stretching vibrations of CH_2_ groups in the benzene ring is observed at 2921 cm^−1^ and in the aliphatic chain at 2853 cm^−1^. Deformation vibrations of -CH_2_ and -CH_3_ groups appear at 1375 cm^−1^. In the 700–900 cm^−1^ region, absorption bands at 869 cm^−1^, 817 cm^−1^, and 746 cm^−1^ are characteristic of aromatic hydrocarbon vibrations, while the band at 721 cm^−1^ indicates the deformation vibrations of alkane chains.

2. Modifying additives:

– ***AS-2*** [25]—a product of the interaction between bottom residues of petrochemical production KON-92 and urea, representing a mixture of amines with the general formula (molecular weight of 237 a.m.u).

R′-NH_2_, R′-NH-R″,

where R′ is n-butyl, and R″ is 2-ethyl-2-hexenyl.

**Figure 2:**
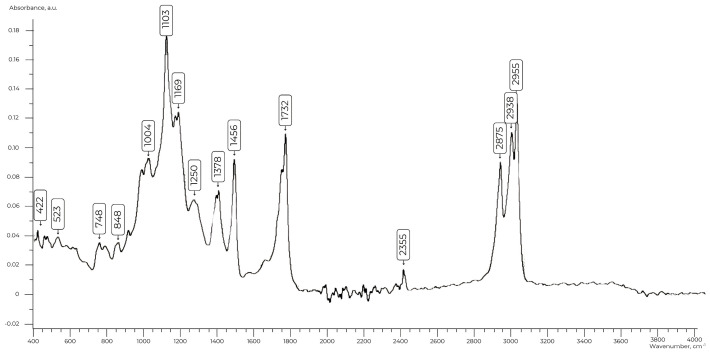
IR spectrum of ***AS-2***.

When analyzing the IR spectra of the obtained compounds (Figure 2), a series of peaks were recorded in the wave region of 700–800 cm^−1^, which corresponds to -NH_2_ vibrations of primary amines, as well as specific absorption bands associated with vibrations of N-H bonds in the region of 1100–1200 cm^−1^ (1103 cm^−1^ and 1169 cm^−1^). The subsequent peaks of 1456 cm^−1^ and 1378 cm^−1^ indicate deformation vibrations of -CH_3_ and -CH_2_ groups. A distinct peak in the region of 1748 cm^−1^ is characteristic of the C=O stretching vibrations of the carbonyl group (1650–1780 cm^−1^). Absorption in the region of 2800–3000 cm^−1^ appeared as two peaks at 2955 cm^−1^ and 2938 cm^−1^ and reflects the C-H stretching vibrations in methyl and methylene groups.

**Figure 3:**
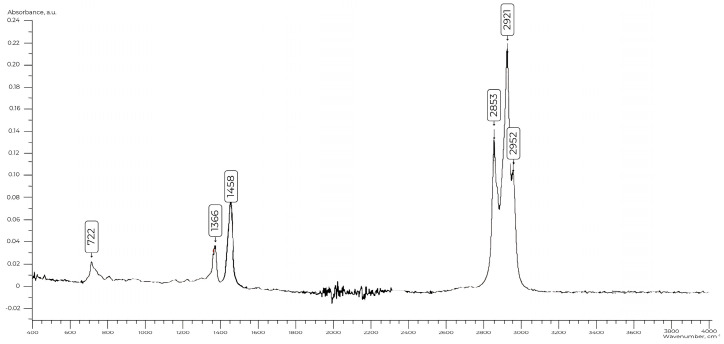
IR spectrum of ***AG-4I***.

– ***AG-4I***—waste sealing liquid, consisting of high-molecular polyisobutylene (PIB) and petroleum oils (molecular weight—5400 a.m.u), produced by “Germetika Research and Production Company”, Moscow, Russia. In the IR spectra of waste sealing liquid (Figure 3), absorption bands corresponding to deformation vibrations of C-H bonds in the region of 722 cm^−1^ are observed.

The doublet at 1366 and 1376 cm^−1^ is attributed to the symmetrical deformation vibrations of the methyl groups. The splitting of the peaks is due to resonance and is characteristic of a dimethyl-substituted chain. The bands observed in the regions of 2853, 2921, and 2952 cm^−1^ are associated with the stretching vibrations of the -CH_2_ groups.

3. The following materials were used as mineral fillers:

– Crushed stone sourced from the “KazRosResurs” deposit in Kazakhstan, with its chemical composition detailed in Table 1 (hereinafter referred to as KS).

– Crushed stone from the “Korneevka” deposit in Kazakhstan, with its chemical composition provided in Table 2 (hereinafter referred to as KA).

### 2.2. Method of Preparation of Modified Bituminous Compositions

The modification of the bitumen binder was performed by dosing the additive into bitumen heated to 130 °C for 30 min with constant mixing in an automatic unit, varying the quantitative content of the modifier in the bitumen from 0.5 to 3.0 g/dm^3^. Mixing and heating of the compositions continued for 40 min to achieve equilibrium physicochemical characteristics (surface tension and contact angle), which were established earlier [26] for binary “bitumen-additive” compositions.

The ternary bitumen compositions were prepared by stepwise dosing of modifiers. At the first stage, a solution of sealing liquid (C = 0.5 ÷ 3.0 g/dm^3^) was introduced into the heated binder, and then the composition was mixed in a thermostat mode (130 °C) for 40 min. At the second stage, ***AS-2*** (C = 0.5 ÷ 3.0 g/dm^3^) was introduced into the binary system “bitumen-***AG-4I***” with a fixed amount of sealant. The mixture was continuously stirred and maintained at a constant temperature for 40 min to ensure the system reached an equilibrium state.

### 2.3. Methodology for Determining the Adhesion Efficiency of Modifiers Based on the Mass of Exfoliated Bitumen

The standard method for assessing the adhesion of bitumen to mineral filler particles involves visually evaluating the area of bitumen binder detachment from the surface of crushed stone when exposed to water [24]. Visual assessment does not allow characterizing the adhesion index with sufficient accuracy; therefore, in this work, we used a quantitative method for determining the adhesive efficiency of modifiers in the bitumen composition, which allowed us to determine the adhesive strength of the bitumen bond to the surface of the mineral material [27]. The essence of the method and calculations of the adhesion index of modified bitumen *X*, as well as the adhesive efficiency *A*, are described in detail by us in the work [26].

### 2.4. Methodology for Determining of the Surface Tension of Bituminous Compositions

The measurement of ***σ_l-g_*** (t = 130 °C) was performed using the Easy Drop method on an automatic setup of the ACAM series (Apex Instruments Co. Pvt. Ltd., Jadavpur Kolkata, India). A 0.25 mL dosing syringe (Gastight, 1725 RN, Hamilton, Hamilton Central Europe S.R.L., Bucharest, Romania) was used to form the drop. This method is described in detail by us in the work [26].

### 2.5. Methodology for Measuring of the Contact Angle

Measurement of contact angle (t = 130 °C) was carried out using an automatic system for measuring the dynamic contact angle and free surface energy of the ACAM. This method is described in detail by us in the work [26].

### 2.6. Method of ProbabilisticDeterministic Planning

In present article, the effect of the concentration of two additives (surfactant ***AS-2*** and polymer ***AG-4I***) for two different grades of crushed stone on the work of adhesion and on the adhesive effectiveness of additives in triple system “bitumen–***AG-4I***–***AS-2***” was studied by the method of probabilistic deterministic planning (PDP). A detailed description of the PDP method, including its comparison with the more widely known Taguchi method, is given in [28].

A 6 × 6 experiment plan was developed for each of two studied grades of crushed stone. In experiments, two parameters varied at six levels:

– Concentration of polymer ***AG-4I*** (***C_AG-4I_***, g/dm^3^) taking the following values: 0.5, 1.0, 1.5, 2.0, 2.5, or 3.0;
– Concentration of surfactant ***AS-2*** (***C_AS-2_***, g/dm^3^) taking the following values: 0.5, 1.0, 1.5, 2.0, 2.5, or 3.0.

## 3. Results and Discussion

### 3.1. Adhesion Properties of Binary Systems “BitumenAdditive” in Relation to the Surface of Mineral Filler

**Figure 4:**
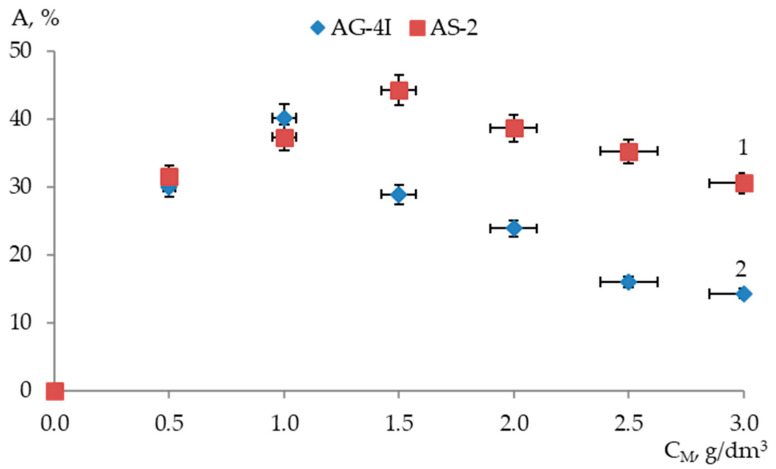
Adhesion efficiency of modifiers in binary bitumen compositions relative to “KazRosResurs” crushed stone surface: 1—***AG-4I***; 2—***AS-2***.

The dependence of the change in the adhesive efficiency of modifiers in the composition of the bitumen binder in relation to the surface of the “KazRosResurs” crushed stone on their quantitative content in binary compositions is shown in Figure 4.

As the content of ***AG-4I*** increased to 1.5 g/dm^3^ in binary bitumen compositions, an increase in the adhesive efficiency of the modified binder in relation to the surface of the “KazRosResurs“ crushed stone was observed. The maximum adhesion efficiency of the modified bitumen (*A_KS_* = 44.23%) was ensured at ***C_AG-4I_*** = 1.5 g/dm^3^ (Figure 4). Introduction of ***AS-2*** into the binder also intensified the adhesion of the modified bitumen to the crushed stone surface although to a lesser extent compared to ***AG-4I***. Thus, in the binary system “bitumen–***AS-2***”, the maximum adhesive efficiency (*A_KS_* = 40.17%) was recorded at a lower additive content (***C_AS-2_*** = 1.0 g/dm^3^). Further dosing of additives in bitumen (***C_AG-4I_*** > 1.5 g/dm^3^; ***C_AS-2_*** > 1.0 g/dm^3^) contributed to a continuous decrease in the adhesive efficiency of the binary compositions down to 30.56% (***C_AG-4I_*** = 3.0 g/dm^3^) and 14.30% (***C_AS-2_*** = 3.0 g/dm^3^).

**Figure 5:**
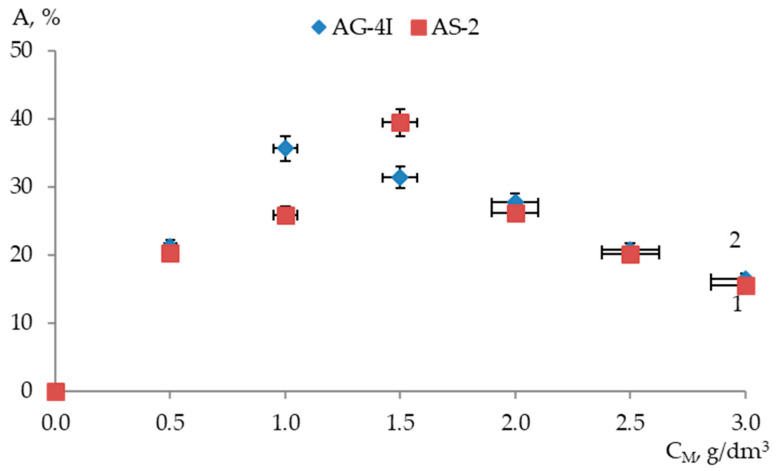
Adhesion efficiency of modifiers in binary bitumen compositions relative to “Korneevka” crushed stone surface: 1—***AG-4I***; 2—***AS-2***.

Changes in the adhesion efficiency of the modifiers in the composition of binary systems in relation to another type of crushed stone (“Korneevka”) reflect the dependencies presented in Figure 5.

On the surface of another variety of crushed stone “Korneevka”, the ability of modifiers to increase the adhesive strength of the binder with the hard surface of the mineral filler decreased (Figure 5). Thus, on the surface of crushed stone “Korneevka”, the adhesive efficiency reached a maximum (*A_KA_* = 39.45%) in the presence of 1.5 g/dm^3^ of ***AG-4I***, which is 4.78% lower than on the surface of crushed stone “KazRosResurs”. For the binary composition “bitumen–***AS-2***” a shift in the extremum in the indicators of adhesive efficiency to the region of lower additive contents was characteristic: the maximum value of *A_KA_* (35.61%) was recorded at ***C_AS-2_*** = 1.0 g/dm^3^, which is 4.56% less than the adhesion efficiency of the isoconcentration composition on the surface of crushed stone “KazRosResurs”. Outside the specified concentration ranges, a decrease in the adhesive efficiency and practically identical *A* values were observed for both binary compositions with the same additive content.

### 3.2. Adhesion Properties of Triple Systems “Bitumen-***AG-4I***-***AS-2***” in Relation to the Surface of Mineral Filler

**Figure 6:**
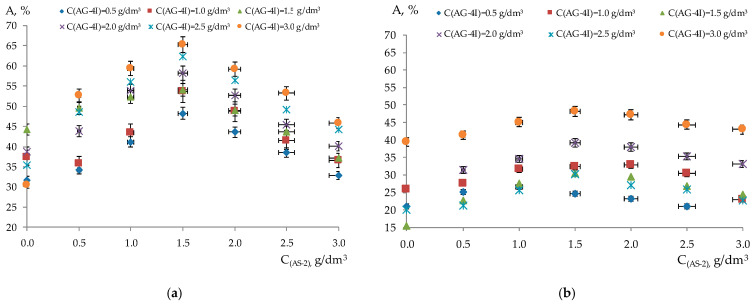
Adhesion efficiency of modifier with fixed ***AG-4I*** content in ternary system relative to crushed stone surface: (**a**) “KazRosResurs”; (**b**) “Korneevka”.

The adhesion efficiency values presented for the triple compositions (Figure 6) demonstrate that the inclusion of ***AS-2*** in bitumen compositions containing a polyisobutylene solution enhanced the adhesive performance of the modified binder on the surfaces of two types of mineral fillers.

The greatest effect of ***AS-2*** on the increase in adhesive efficiency was recorded at ***C_AG-4I_*** = 3.0 g/dm^3^ and ***C_AS-2_*** = 1.5 g/dm^3^ in a triple bitumen composition. In this concentration mode, the maximum adhesive efficiency (A_max_ = 65.18%) was recorded on the surface of the “KazRosResurs” crushed stone, as well as in relation to the “Korneevka” crushed stone (A_max_ = 48.11%). The concentration of ***AS-2*** to 3.0 g/dm^3^ and a decrease in the content of ***AG-4I*** to 0.5 g/dm^3^ in the bitumen compositions contributed to a decrease in the values of adhesive efficiency in relation to two types of crushed stone (A_min_ = 32.87%, “KazRosResurs”; A_min_ = 21.00%, “Korneevka”).

To identify the mechanism of changes in the adhesive effectiveness of bitumen under the influence of modifiers, a cycle of physicochemical studies was carried out, which included measuring of surface tension and the contact angle.

Previously, we identified the localization of the introduced nitrogen-containing surfactant, as well as surfactants released from the micellar structure of bitumen under the influence of a limited concentration of ***AG-4I*** [26]. The key factor in achieving the wetting effect of additives with respect to mineral fillers is the localization of their polar groups at the active sites on the mosaic-like solid surface of the crushed stone.

### 3.3. Study of Adhesive and Cohesive Strength of Binary “BitumenAdditive”from the Concentration of Additives by the Thermodynamic Method

The influence of the concentration of the ***AS-2*** modifier on the changes occurring at the interphase boundaries of bitumen with two types of mineral fillers and air is presented in Table 3.

Based on a comparison of the obtained values of the surface tension of modified bitumen solutions (***σ_l-g_***) and the contact angle of wetting of the crushed stone surface (*θ*), the following can be stated:

1. The addition of ***AS-2*** into bitumen intensifies the wetting process of the crushed stone surface, as indicated by the reduction in the contact angle *θ*. The maximum wetting effect of ***AS-2*** (minimum *θ* in Table 3) occurred at its concentration in bitumen of 1.0 g/dm^3^. The contact angle decreased by 9.44° compared to unmodified bitumen and amounted to 130.66° (“KazRosResurs”); for another type of crushed stone (“Korneevka”)—by 7.73° (θ = 134.38°). A further increase in the ***AS-2*** content (C > 1.0 g/dm^3^) was accompanied by an increase in contact angles with their subsequent stabilization at the level of 138–141° (***C_AS-2_*** = 2.50–3.0 g/dm^3^).

2. The changes in the *θ* values do not exhibit symmetry with the variations in the surface tension of bitumen at the “bitumen–air” interface across the entire range of additive concentrations studied: we recorded a decrease in the ***σ_l-g_*** values (***C_AS-2_*** ≤ 1.0 g/dm^3^) followed by their continuous growth in the region of increased contents (***C_AS-2_*** > 1.0 g/dm^3^).

These results prove the ability of ***AS-2*** (C_***AS-2***_ ≤ 1.0 g/dm^3^) to be adsorbed both on the solid surface of crushed stone and on the bitumen–air interface. As a result of adsorption, the values of *σ_s-l_* and ***σ_l-g_*** decrease, leading to improved wetting of mineral fillers within this concentration range, in accordance with the well-known Young’s equation [20].

According to the obtained data (Table 3), the introduction of ***AS-2*** into bitumen up to 1.0 g/dm^3^ was accompanied by a decrease in the intermolecular interaction forces in the volume; a decrease in the work of cohesion in the binary composition “bitumen–***AS-2***” with an increase in the additive concentration from 0 to 1.0 g/dm^3^ was 8.52 mN/m. In the same series of changes in the concentration of ***AS-2***, judging by the values of the equilibrium work of adhesion, an increase in the strength of bitumen fixation on the surface of mineral fillers was observed; the values of the work of adhesion with an increase in the concentration of ***AS-2*** from 0 to 1.0 g/dm^3^ increased from 9.43 to 10.32 mN/m (crushed stone “KazRosResurs”) and from 8.51 to 10.87 mN/m (crushed stone “Korneevka”). However, beyond the threshold of this concentration section (C_***AS-2***_ > 1.0 g/dm^3^), an opposite trend was noted—an increase in cohesive interactions in the volume of the bitumen binder accompanied by a slight decrease in the work of adhesion (ΔW_A_ = 0.34–1.21 mN/m) necessary for breaking a unit area of the interphase surface layer. This is due to the fact that dosing the cation-active additive over 1.0 g/dm^3^ contributed to the concentration of ***AS-2*** in the volume of the dispersed system, but not at the interphase boundary with air. This is evidenced by the continuous shift of surface tension to the region of large values (Table 3). Under the influence of associative transformations of ***AS-2*** in the range of increased additive concentrations (from 1.0 to 3.0 g/dm^3^), the value of the work of cohesion increased from 72.48 to 88.20 mN/m.

The obtained values of the work of adhesion in the studied region of increased ***AS-2*** contents are due to the different intensity of changes, on the one hand, of intermolecular interactions in the volume of bitumen and, on the other hand, of the wetting effect in relation to the solid phase dispersion of crushed stone: the increase in ***σ_l-g_*** was greater than the decrease in ***cosθ***. This indicates the preference for migration of surfactants from the volume to the more energetically favorable surface of the crushed stone than to the “bitumen–air” boundary. Thus, if at C_***AS-2***_ = 2.0 g/dm^3^, no surfactant molecules are present at the “bitumen–air” interphase boundary (*σ*_l-g_ at the level of the unmodified binder is 40.18 mN/m), then they are present on the “bitumen–crushed stone” surface (***cosθ*** is higher than that of bitumen in the absence of ***AS-2***), as well as beyond this concentration (***C_AS-2_*** > 2.0 g/dm^3^).

The extreme nature of change in ***σ_l-g_*** and ***cosθ*** was also observed when introducing the sealing liquid ***AG-4I*** into the bitumen (Table 4). However, the ***AG-4I*** additive was characterized by a more significant decrease in the surface tension (***σ_l-g_***) and contact angle (*θ*), along with a shift of their minimum values to higher concentrations (***C_AG-4I_*** = 1.5 g/dm^3^). Thus, as the ***AG-4I*** content in bitumen increased from 0 to 1.5 g/dm³, the values of ***σ_l-g_*** decreased from 40.50 to 34.24 mN/m. In the same range of modifiers concentrations, the amplitude of the decrease in the contact angle of wetting the surface of “KazRosResurs” crushed stone was 13.65°; on the other type of mineral filler “Korneevka”, it was 10.17°. The obtained results prove that bituminous surfactants [26], delocalized under the influence of a limited concentration of sealing liquid (***C_AG-4I_*** > 1.5 g/dm^3^), exhibited greater surface activity on the interface surfaces of the binder with air (***σ_l-g_***) and crushed stone (*σ_s-l_*) than the introduced modifier ***AS-2***. As a result, the wetting effect of ***AG-4I*** in relation to the mineral filler is higher.

The observed difference with ***AS-2*** (Table 3) is associated with both the different architecture of the additive molecules (composition, structure, etc.) and with the factor of localization of the polar groups of the surfactant on the chemically heterogeneous surface of the crushed stone (Table 1).

A solution of polyisobutylene in mineral oil (***AG-4I***), as we have established earlier [29], in a limited concentration range causes the destruction of large bitumen micro associates with the release of surface-active components included in their composition. Due to surface activity, surfactants are concentrated at the interphase boundaries of the phase separation, causing the observed changes in ***σ_l-g_*** and ***cosθ***. As a result, in this concentration section of the additive (from 0 to 1.5 g/dm^3^), a more significant decrease in cohesive work was recorded in comparison to ***AS-2*** (the ***W_K_*** value decreased by 12.52 mN/m) with a simultaneous increase in ***W_A_*** from 9.32 to 14.04 mN/m (“KazRosResurs”). On the surface of another mineral filler (“Korneevka”), even with a very small change in the total content of Al_2_O_3_, CaO, Na_2_O, Fe_2_O_3_, TiO_2_ (a decrease of 1.3%), and SiO_2_ (an increase of 1.2%), the wetting activity and, accordingly, the strength of fixation of the same binary compositions with ***AG-4I*** decreased. The increase in the work ofadhesion in the same range of modifier concentrations (from 0 to 1.5 g/dm^3^) was two times less (Δ*W_A_* = 1.63 mN/m) than on the previous crushed stone (Δ*W_A_* = 3.69 mN/m). This is a clear demonstration of the influence of the nature of adsorption centers (strength and activity) on the processes of oleophilization of the surface of mineral fillers and, as a consequence, the adhesive bond of the binder to their surface.

At higher concentrations (***C_AG-4I_*** > 1.5 g/dm³), the structuring effect of the intermolecular network formed by polyisobutylene, the second component of ***AG-4I***, was intensified. This polymer association process was accompanied by the spontaneous migration of surfactant molecules from the “bitumen–air” interface into the bulk of the binder’s dispersed system, resulting in a continuous increase in ***σ_l-g_*** (Table 4). The formation of mixed spatial structures contributed to the strengthening of intermolecular interactions within the composition and, as a consequence, to the increase of ***W_K_***. Thus, at ***C_AG-4I_*** = 2.5 g/dm^3^, the work of cohesionincreased by 9.20 mN/m compared to unmodified bitumen and amounted to 90.02 mN/m. It should be noted that the presence of surface-active components is retained on the “bitumen–crushed stone” surface in the area of increased ***AG-4I*** contents, but in smaller quantities due to the development of association processes, which worsens the oleophilization of crushed stone (repeated jump *θ*). A smaller decrease in the surface tension of the solid surface in the concentration section of the additive from 1.5 to 3.0 g/dm^3^ was accompanied by a decrease in the ***W_A_*** from 14.04 to 11.12 mN/m (“KazRosResurs”) and from 11.30 to 9.55 mN/m (“Korneevka”).

### 3.4. Study of Adhesive and Cohesive Strength of Triple Systems “Bitumen–***AG-4I***–***AS-2***” from the Concentration of Additives by the Thermodynamic Method

The experimental values of surface tension (σ_l-g_^ex^^*^) and the calculated values (σ_l-g_^c^^*^) for the mixed compositions, which include the simultaneous presence of ***AG-4I*** and ***AS-2***, are presented in Table 5.

(3) $$\sigma_{l-g}^{C*}=\sigma_{l-g}^{0}-\left(∆\sigma_{AG-4I}+∆\sigma_{AS-2}\right),$$

where **Δ*σ_AG-_*_4*I*_** and **Δ*σ_AS-_*_2_** are the changes in surface tension in the binary system relative to the virgin bitumen (***σ*^0^*_l-g_***).

On an energetically homogeneous liquid bitumen surface bordering air, the maximum decrease in the surface tension ***σ_l-g_*** was observed at the same concentrations as in binary “bitumen-additive” compositions (Table 3 and Table 4). With the combined introduction of ***AS-2*** (1.0 g/dm^3^) and ***AG-4I*** (1.5 g/dm^3^), the surface tension decreased by 10.04 mN/m compared to virgin bitumen (*σ*^0^*_l-g_* = 81.00 mN/m). The resulting change in surface tension Δ*σ_l-g_* was determined by the additive contribution of **Δ*σ_AG-4I_*** and **Δ*σ_AS-2_*** (Table 5), indicating the absence of intermolecular interactions and volumetric complexities at the “bitumen–air” interface (*σ^C^_l-g_* and *σ^ex^_l-_*_g_ are virtually identical). The observed fluctuations from the additivity rule in the region of elevated ***AG-4I*** contents (***C_AG-4I_*** > 2.0 g/dm^3^) are obviously associated with the screening effect of one of the two components present at the “bitumen–air” interface.

**Figure 7:**
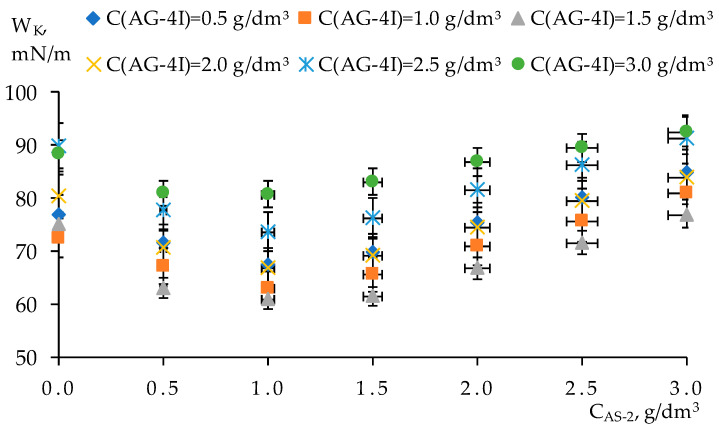
Dependence of the work of cohesion on the content of ***AS-2*** in compositions with a fixed content of ***AG-4I***: 0.5 g/dm^3^; 1.0 g/dm^3^; 1.5 g/dm^3^; 2.0 g/dm^3^; 2.5 g/dm^3^; 3.0 g/dm^3^.

The maximum surface activity of additives corresponds to the greatest effect of bitumen destructuring (Figure 7). With the content of additives in bitumen (***C_AS-2_*** = 1.0 g/dm^3^; ***C_AG-4I_*** = 1.5 g/dm^3^), a minimum of work was noted and spent on breaking the bulk phase of bitumen over a section equal to a unit area (***W_K_*** = 60.92 mN/m). In the region of lower concentrations of ***AS-2*** (***C_AS-2_*** ≤ 1.0 g/dm^3^) at all contents of ***AG-4I*** in the ternary compositions, the decrease in the cohesive work is a detector of dissociative processes occurring during the reconstruction of the dispersed system of bitumen under their influence. The depth of these processes was greater than in the binary compositions. Thus, in the composition with the content of additives (***C_AS-2_*** = 0.5 g/dm^3^; ***C_AG-4I_*** = 1.5 g/dm^3^), the cohesive work was lower by 5.40 mN/m than the ***W_K_*** of the binary composition “bitumen–***AG-4I***” (***C_AG-4I_*** = 1.5 g/dm^3^) and by 9.36 mN/m in comparison with the binary composition “bitumen–***AS-2***” (***C_AS-2_*** = 0.5 g/dm^3^).

In the region of increased concentrations of additives (***C_AS-2_*** > 1.0 g/dm^3^; ***C_AG-4I_*** > 1.5 g/dm^3^), the increase in the work of cohesion demonstrated in Figure 7 serves as a detector of associative transformations. The greatest effect of bitumen structuring (***W_K_*** = 92.56 mN/m) was observed at the maximum content of additives (***C_AS-2_*** = 3.0 g/dm^3^; ***C_AG-4I_*** = 3.0 g/dm^3^) in mixed composition compositions. This value of cohesive work exceeded ***W_K_*** of unmodified bitumen by 11.66 mN/m. In comparison with binary isoconcentration compositions, the increase in ***W_K_*** was 9.48 mN/m (***C_AS-2_*** = 3.0 g/dm^3^) and 4.36 mN/m (***C_AG-4I_*** = 3.0 g/dm^3^).

Table 6 presents the experimental measurements of the contact angle on the energetically heterogeneous surfaces of two types of crushed stone using the “bitumen–AG-4I–***AS-2***” compositions.

As the experimental data demonstrate, the incorporation of limited concentrations of ***AS-2*** (***C_AS-2_*** ≤ 1.5 g/dm^3^) into the bitumen composition with a polyisobutylene solution enhanced the wetting processes for both the “KazRosResurs” and “Korneevka” crushed stone. Adding ***AS-2*** in this concentration range shifted the *θ* to a range of lower values and reached a minimum at ***C_AS-2_*** = 1.5 g/dm^3^ and ***C_AG-4I_*** = 1.5 g/dm^3^: the composition “bitumen–AG-4I–***AS-2***” provides a reduction in the *θ* by 15.04° (“KazRosResurs”) and by 14.07° (“Korneevka”). This indicates that in this range of modifier concentrations (C_M_ ≤ 1.5 g/dm^3^), a deeper decrease in the contact angle value occurs as a result of the joint contribution of ***AS-2*** and ***AG-4I*** to the decrease in ***σ_l-g_*** and *σ_s-l_*.

**Figure 8:**
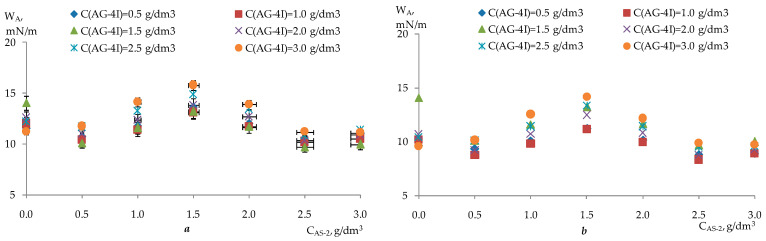
Dependence of the work of adhesion on the content of ***AS-2*** in compositions with a fixed concentration of ***AG-4I*** in relation to crushed stone: (**a**) “KazRosResurs”; (**b**) “Korneevka”.

With a further increase in the ***AS-2*** content (***C_AS-2_*** > 1.5 g/dm^3^), a gradual deterioration in the wetting of mineral fillers occurred. As the ***AS-2*** concentration increased (from 1.5 to 3.0 g/dm^3^), and the *θ* increased by 10.0° (from 127.85° to 137.85°) on the surface of “KazRosResurs” crushed stone and by 8.8° in relation to the surface of “Korneevka” crushed stone (from 132.98° to 141.78°). As for the influence of the concentration factor of ***AG-4I***, as shown by the experimental data (Table 6), each additional dosing of 0.5 g/dm^3^ of sealing liquid into bitumen did not have a significant effect on *θ* (with a fixed content of ***AS-2***). This indicates the screening effect of the combined presence of crushed stone on the energetically non-uniform surface and the competition for adsorption centers. The values of *θ* had only minor deviations (±2.00°) from the initial value determined by the content of 0.5 g/dm^3^ ***AG-4I***. Thus, with a 6-fold increase in the ***AG-4I*** concentration (3.0 g/dm^3^) in the “bitumen–AG-4I–***AS-2***” system with a fixed ***AS-2*** concentration (***C_AS-2_*** = 1.5 g/dm^3^), the contact angle remained practically at the same level—128.04° (“KazRosResurs”) and 130.98° (“Korneevka”)—as with ***C_AG-4I_*** = 0.5 g/dm^3^. It follows from this that in the composition “bitumen–AG-4I–***AS-2***” with ***C_AS-2_*** = 1.5 g/dm^3^, the wetting of the crushed stone surface was the maximum at any content of ***AG-4I*** and practically did not change, but at the same time, another factor changed—the cohesion. As the content of ***AG-4I*** in the composition of the mixed composition increased, the intermolecular interaction inside the bitumen invariably increased and reached maximum values at ***C_AG-4I_*** = 3.0 g/dm^3^ (Figure 7). Thus, the conjugation of the wetting and cohesive factors reached the maximum possible values at ***C_AG-4I_*** = 3.0 g/dm^3^ and ***C_AS-2_*** = 1.5 g/dm^3^ in the bitumen binder. This led, in accordance with the combined Dupré–Young equation, to the highest works of adhesion of the binder with mineral fillers in the entire range of the studied ratios of additives (Figure 8).

The equilibrium work of adhesion ***W_A_*** had a value of 15.79 mN/m on the surface of “KazRosResurs” mineral filler and 14.13 mN/m on the “Korneevka” crushed stone. With this optimal ratio of components (***C_AG-4I_*** = 3.0 g/dm^3^ and ***C_AS-2_*** = 1.5 g/dm^3^), the amplitude of the increase in the work required to rupture the interphase surface layer was 6.47 mN/m (“KazRosResurs” crushed stone) and 5.62 mN/m (“Korneevka” crushed stone) relative to virgin bitumen (Table 3 and Table 4).

This explains the fact that the previously described adhesive efficiency (A, %) of modified bitumen compositions had maximum values (Figure 6) precisely in these concentration modes of modifiers.

Thus, the addition of the studied additives in optimal ratio led to an improvement in the adhesion of the bitumen composition to the surface of the crushed stone, which led to an increase in the durability of asphalt pavements.

### 3.5. Modeling the Effect of Concentrations of ***AG-4I*** and ***AS-2*** on the Thermodynamic Work of Adhesion and the Adhesive Efficiency of Triple Bitumen Compositions

Table 7 shows the experiment plan and the results of indirect measurements of the work of adhesion (*W_A_KS_* and *W_A_KA_*, mN/m) and of the adhesive effectiveness of the additive (*A_KS_* and *A_KA_*, %). Parameters *W_A_KS_* and *A_KS_* describe adhesive properties of the triple system for the “KazRosResurs” grade of crushed stone, while *W_A_KA_* and *A_KA_* are for the “Korneevka” grade of crushed stone, accordingly.

**Figure 9:**
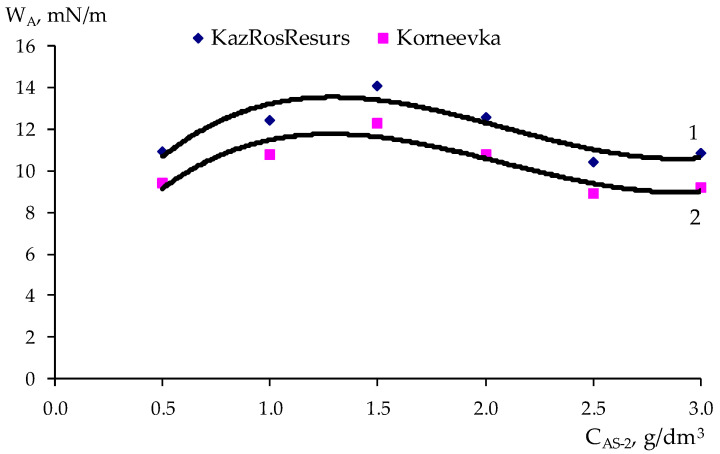
Effect of the ***AS-2*** concentration on the work of adhesion for two grades of crushed stone: 1—“KazRosResurs”; 2—“Korneevka”.

**Figure 10:**
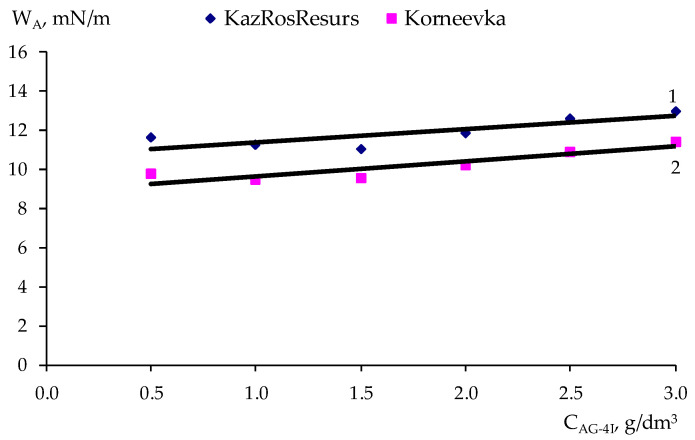
Effect of the ***AG-4I*** concentration on the work of adhesionfor two grades of crushed stone: 1—“KazRosResurs”; 2—“Korneevka”.

**Figure 11:**
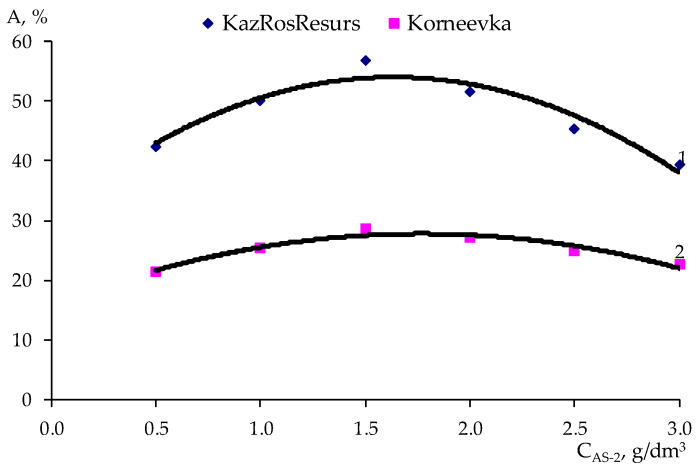
Effect of the ***AS-2*** concentration on the adhesive effectiveness of the additive for two grades of crushed stone: 1—“KazRosResurs”; 2—“Korneevka”.

**Figure 12:**
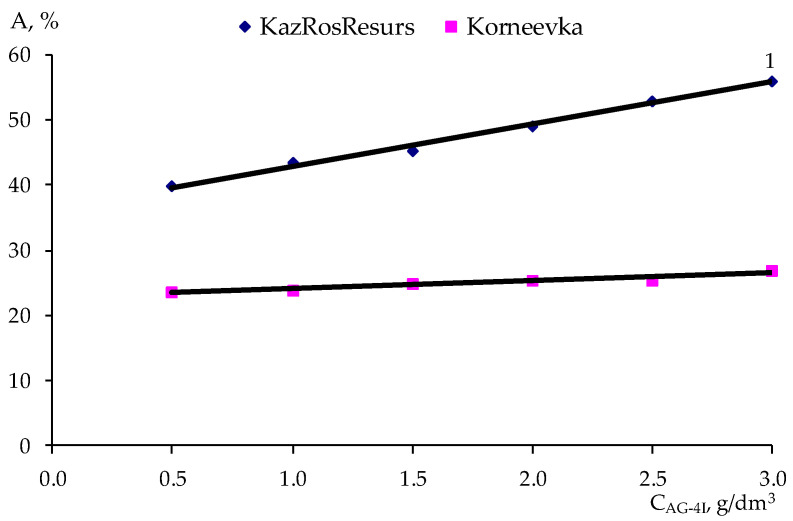
Effect of the ***AG-4I*** concentration on the adhesive effectiveness of the additive for two grades of crushed stone:1—“KazRosResurs”; 2—“Korneevka”.

In accordance with the method of PDP [28], a sample of the experimental data (Table 7) was carried out on point graphs (Table 8 and Table 9), and partial dependency graphs were generated, as shown in Figure 9, Figure 10, Figure 11 and Figure 12.

The partial dependences presented in Figure 9, Figure 10, Figure 11 and Figure 12 were approximated by polynomials of various orders. Using the obtained polynomials, two-factor mathematical models were constructed based on the generalized Protodiakonov equation [28], taking into account the combined effect of the concentration of ***AG-4I*** and the concentration of ***AS-2*** on the work of adhesionfor crushed stone of the “KazRosResurs” grade (Equation (4)) and for crushed stone of the “Korneevka” grade (Equation (5)), as well as for the adhesive activity of additives for crushed stone of the “KazRosResurs” grade (Equation (6)) and for crushed stone of the “Korneevka” grade (Equation (7)):

(4) $$W_{A\_KS}=\frac{\left(1.4325C_{AS\text{-}2}^{3}-8.9941C_{AS\text{-}2}^{2}+16.0550+4.7166)(0.6696C_{AG\text{-}4I}+10.70\right)}{11.87},\;mN/m\;$$

(5) $$W_{A\_KA}=\frac{\left(1.3442C_{AS\text{-}2}^{3}-8.4030C_{AS\text{-}2}^{2}+14.9070C_{AS\text{-}2}+3.6313)(0.7573C_{AG\text{-}4I}+8.8816\right)}{10.21},\;mN/m$$

(6) $$A_{KS}=\frac{\left(-8.5854C_{AS\text{-}2}^{2}+28.058C_{AS\text{-}2}+31.083)(6.4692C_{AG\text{-}4I}+36.311\right)}{47.63},\;\%$$

(7) $$A_{KA}=\frac{\left(-3.8081C_{AS\text{-}2}^{2}+13.4730C_{AS\text{-}2}+15.844)(1.2426C_{AG\text{-}4I}+22.809\right)}{24.98},\;\%$$

The reliability of the obtained mathematical models was estimated by calculating the coefficients of nonlinear multiple correlation [28]. The minimum coefficient of nonlinear multiple correlation among the proposed mathematical models came out to 0.87. The significance of the obtained coefficients of nonlinear multiple correlation was confirmed using the Fisher criterion.

**Figure 13:**
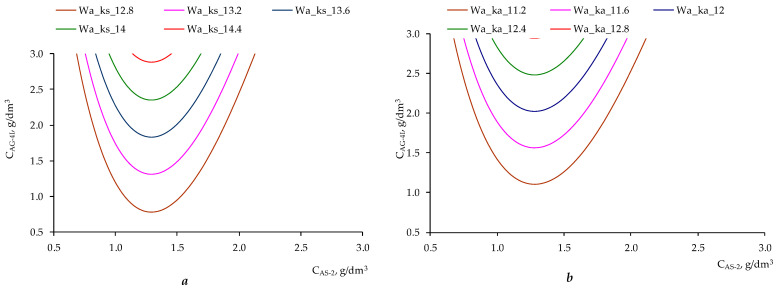
Nomograms for achieving the desired work of adhesion for various ratios between concentrations of ***AS-2*** and ***AG-4I***: (**a**) for crushed stone of the “KazRosResurs” grade; (**b**) for crushed stone of the “Korneevka” grade.

**Figure 14:**
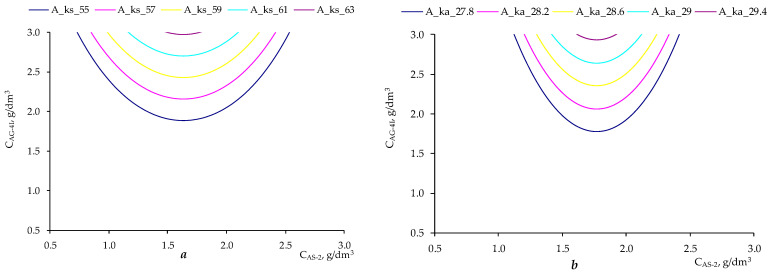
Nomograms for achieving the desired adhesive effectiveness of the additive for various ratios between concentrations of ***AS-2*** and ***AG-4I***: (**a**) for crushed stone of the “KazRosResurs” grade; (**b**) for crushed stone of the “Korneevka” grade.

Nomograms were constructed based on the obtained two-parameter mathematical models (Figure 13 and Figure 14). The constructed nomograms (Figure 13) make it possible to achieve the required work of adhesion value for crushed stone of the “KazRosResurs” grade (*W_A_KS_* takes the values 12.8, 13.2, 13.6, 14.0, and 14.4 mN/m, respectively) and for crushed stone of the “Korneevka” grade (*W_A_KA_* takes the values 11.2, 11.6, 12.0, 12.4, and 12.8 mN/m, respectively) at different ratios of concentrations of ***AG-4I*** (***C_AG-4I_***, g/dm^3^) and ***AS-2*** (***C_AS-2_***, g/dm^3^).

Figure 14 shows nomograms that allow achieving the required value of the adhesive effectiveness of the additive for crushed stone of the “KazRosResurs” grade (*A_KS_* takes values 55, 57, 59, 61 and 63%, respectively) and for crushed stone of the “Korneevka” grade (*A_KA_* takes values 27.8, 28.2, 28.6, 28.0, and 28.4%, respectively) at different ratios of concentrations of ***AG-4I*** (***C_AG-4I_***, g/dm^3^) and ***AS-2*** (***C_AS-2_***, g/dm^3^).

The constructed nomograms (Figure 13 and Figure 14) facilitate the selection of optimal additive concentrations optimal additives concentrations to achieve the desired work of adhesion and adhesive effectiveness of the additive in the triple system “bitumen–***AG-4I***–***AS-2***”.

## 4. Conclusions

1. Adhesion–cohesion processes in modified bitumen systems are limited by the wetting effect of mineral fillers and the intensity of intermolecular forces in the condensed phase of the binder.
2. In the binary composition “bitumen–***AG-4I***”, the minimum cohesive work (***W_K_*** = 72.48 mN/m), the highest adhesive efficiency (*A_KS_* = 44.23%; *A_KA_* = 39.45%), and the maximum thermodynamic work of adhesion (*W_A(KS)_* = 14.04 mN/m; *W_A(KA)_* = 11.30 mN/m) on the surface of two types of mineral fillers werefixed at the same concentration (***C_AG-4I_*** = 1.5 g/dm^3^) as the smallest contact angle (*θ_KS_* = 126.45°; *θ_KA_* = 131.94°).
3. In the binary composition “bitumen–***AS-2***”, the minimum cohesive work (***W_K_*** = 72.48 mN/m), the highest adhesive efficiency (*A_KS_* = 40.17%; *A_KA_* = 35.61%), and the maximum thermodynamic work of adhesion (*W_A(KS)_* = 12.68 mN/m; *W_A(KA)_* = 10.87 mN/m) on the surface of both types of mineral fillers were also revealed at the maximum wetting effect (*θ_KS_* = 130.66°; *θ_KA_* = 134.38°) but in the region of lower additive concentrations (***C_AS-2_*** = 1.0 g/dm^3^).
4. The addition of ***AS-2*** into bitumen compositions with polyisobutylene solution increases adhesion efficiency and thermodynamic work of adhesion in relation to the filler surface. The adhesion efficiency and thermodynamic work of adhesion in the “bitumen-***AG-4I***-***AS-2***” system reached their maximum at ***C_AG-4I_*** = 3.0 g/dm^3^ and ***C_AS-2_*** = 1.5 g/dm^3^. In these concentration ranges, the composition achieved the maximum increase in adhesion efficiency (*A_KS_* = 65.18%; *A_KA_* = 48.11%) and the greatest increase in thermodynamic work of adhesion (*W_A(KS)_* = 15.79 mN/m; *W_A(KA)_* = 14.13 mN/m).
5. Based on the results of mathematical modeling, two-factor nomograms were developed, enabling the solution of practical problems, particularly the optimization of bitumen–mineral compositions with specified adhesive properties.
6. A comparative analysis of the two methods for determining the adhesive–cohesive interactions of bitumen in the presence of additives has shown that the thermodynamic approach is fundamental to justify the obtained modification results.
7. One of the directions of our further research is the development of a methodology for determining quantitative indicators of cohesive and adhesive fracture of contact surfaces of mineral filler with modified bitumen using computer-optical microscopy, including a method and algorithm for its implementation.

## Figures and Tables

**Table 1:** Chemical composition of the “KazRosResurs” crushed stone.

SiO_2_ %	TiO_2_ %	Al_2_O_3_ %	Fe_2_O_3_ %	CaO %	MgO %	S %	Mn ppm	K_2_O %	Na_2_O %
75.37	0.98	12.04	2.67	0.78	0.31	-	774	5.27	2.58

**Table 2:** Chemical composition of the “Korneevka” crushed stone.

SiO_2_ %	TiO_2_ %	Al_2_O_3_ %	Fe_2_O_3_ %	CaO %	MgO %	Cl %	MnO ppm	K_2_O %	Na_2_O %
76.50	0.90	11.70	2.37	0.38	0.31	0.14	257	5.29	2.40

**Table 3:** Changes in bitumen indicators at the interphase boundaries with air and crushed stone depending on the content of ***AS-2*** in the composition.

*C_Ac-2_*, g/dm^3^	***σ_l-g_***, mN/m	***W_K_***, mN/m	“KazRosResurs”		“Korneevka”	
			*θ*, °	*W_A_*, mN/m	*θ*, °	*W_A_*, mN/m
0	40.50	81.00	140.10	9.32	142.11	8.51
0.5	38.36	76.72	134.88	11.12	138.25	9.59
1.0	36.24	72.48	130.66	12.68	134.38	10.87
1.5	37.54	75.08	132.63	12.01	136.66	10.14
2.0	40.18	80.36	134.60	12.05	138.45	10.05
2.5	44.84	89.68	137.76	11.66	140.83	9.86
3.0	44.10	88.20	137.93	11.47	140.98	9.70

**Table 4:** Changes in bitumen indicators at the interphase boundaries with air and crushed stone depending on the content of ***AG-4I*** in the composition.

***C_AG-4I_***, g/dm^3^	***σ_l-g_***, mN/m	***W_K_***, mN/m	“KazRosResurs”		“Korneevka”	
			*θ*, °	*W_A_*, mN/m	*θ*, °	*W_A_*, mN/m
0	40.50	81.00	140.10	9.32	142.11	8.51
0.5	38.42	76.84	134.55	11.53	138.16	9.61
1.0	36.32	72.64	131.92	11.99	136.08	10.17
1.5	34.24	68.48	126.45	14.04	131.94	11.30
2.0	38.06	76.12	132.11	12.56	136.14	10.66
2.5	45.10	90.20	136.77	12.18	139.96	10.37
3.0	41.54	83.08	136.93	11.22	139.98	9.55

**Table 5:** Surface tension of ternary systems at the boundary with air.

C*_AG-4I_*, g/dm^3^	0.5		1.0		1.5		2.0		2.5		3.0	
C*_AS-2_*, g/dm^3^	*σ*^ex*^, mN/m	*σ*^c^, mN/m	*σ*^ex*^, mN/m	*σ*^c^, mN/m	*σ*^ex*^, mN/m	*σ*^c^, mN/m	*σ*^ex*^, mN/m	*σ*^c^, mN/m	*σ*^ex*^, mN/m	*σ*^c^, mN/m	*σ*^ex*^, mN/m	*σ*^c^, mN/m
0.5	35.72	36.28	33.58	34.18	31.54	32.10	35.38	35.92	38.82	42.96	40.48	39.40
1.0	33.68	34.16	31.52	32.06	30.46	29.98	33.36	33.80	36.82	40.84	40.38	37.28
1.5	34.94	35.46	32.80	33.36	30.72	31.28	34.56	35.10	38.08	42.14	41.56	38.58
2.0	37.65	38.10	35.42	36.00	33.38	33.92	37.28	37.74	40.74	44.78	43.38	41.22
2.5	39.98	42.76	37.80	40.66	35.82	38.58	39.72	42.20	43.14	49.44	44.74	45.88
3.0	42.42	42.02	40.46	39.92	38.36	37.84	42.02	41.66	45.60	48.70	46.28	45.14

**Table 6:** Contact angle of wetting of mineral fillers surface with triple bitumen compositions.

**Crushed Stone “KazRosResurs”**						
*****C_AG-4I_***, g/dm^3^**	**0.5**	**1.0**	**1.5**	**2.0**	**2.5**	**3.0**
*****C_AS-2_***, g/dm^3^**	***θ*, °**	***θ*, °**	***θ*, °**	***θ*, °**	***θ*, °**	***θ*, °**
0	134.55	131.92	126.45	132.11	136.77	136.93
0.5	134.14	133.98	132.87	133.54	134.69	135.48
1.0	130.26	129.73	128.17	129.32	130.03	130.31
1.5	127.85	126.58	125.06	126.55	127.29	128.04
2.0	133.14	132.41	130.75	131.28	132.26	132.68
2.5	137.49	137.24	137.19	137.61	138.18	138.46
3.0	137.85	137.52	137.38	137.91	138.54	139.08
**Crushed stone “Korneevka”**						
*****C_AG-4I_***, g/dm^3^**	**0.5**	**1.0**	**1.5**	**2.0**	**2.5**	**3.0**
*****C_AS-2_***, g/dm^3^**	***θ*, °**	***θ*, °**	***θ*, °**	***θ*, °**	***θ*, °**	***θ*, °**
**0**	138.16	136.08	131.94	136.14	139.96	139.98
0.5	137.98	137.42	136.34	137.59	137.92	138.24
1.0	134.27	133.98	132.19	132.78	133.31	133.94
1.5	132.98	131.55	128.97	129.58	130.39	130.98
2.0	136.83	136.37	134.79	135.5	135.69	136.26
2.5	141.24	141.02	140.29	140.98	141.32	141.67
3.0	141.78	141.53	140.12	141.23	141.74	141.98

**Table 7:** Two-factor plan of experiment at 6 levels to study the effect of concentrations of jointly present additives ***AG-4I*** and ***AS-2*** on the work of adhesion and on the adhesive effectiveness of an additive for two different grades of crushed stone.

Concentrations of Additives		Grade of Crushed Stone			
		“KazRosResurs”		“Korneevka”	
***C_AS-2_***, g/dm^3^	***C_AG-4I_***, g/dm^3^	*W_A_KS_*, mN/m	*A_KS_*, %	*W_A_KA_*, mN/m	*A_KA_*, %
0.50	0.50	10.72	34.22	9.29	21.03
1.00	0.50	11.79	41.15	10.10	25.20
1.50	0.50	13.63	48.19	11.18	26.55
2.00	0.50	12.05	43.57	10.17	24.62
2.50	0.50	10.39	38.60	8.80	23.27
3.00	0.50	11.03	32.87	8.91	21.00
0.50	1.00	10.41	35.69	8.73	20.55
1.00	1.00	11.35	43.45	9.77	24.72
1.50	1.00	13.12	53.64	11.15	27.33
2.00	1.00	11.69	48.71	9.92	25.98
2.50	1.00	10.21	41.38	8.32	23.35
3.00	1.00	10.52	36.53	8.90	20.97
0.50	1.50	10.09	39.47	8.83	21.34
1.00	1.50	11.57	47.23	10.05	25.11
1.50	1.50	13.21	54.08	11.37	28.11
2.00	1.50	11.68	49.03	10.01	27.16
2.50	1.50	9.67	43.65	8.24	24.32
3.00	1.50	9.97	37.17	8.82	23.03
0.50	2.00	10.97	43.81	9.20	21.50
1.00	2.00	12.34	53.88	10.68	24.63
1.50	2.00	13.82	58.18	12.44	29.15
2.00	2.00	12.68	52.65	10.81	28.01
2.50	2.00	10.33	45.44	8.74	25.32
3.00	2.00	10.93	40.07	9.24	23.17
0.50	2.50	11.65	48.51	10.09	21.15
1.00	2.50	13.26	56.03	11.41	25.56
1.50	2.50	14.85	62.20	13.33	30.13
2.00	2.50	13.44	56.47	11.41	27.11
2.50	2.50	10.79	49.18	9.49	25.88
3.00	2.50	11.40	44.29	9.58	22.70
0.50	3.00	11.74	52.65	10.12	22.69
1.00	3.00	14.13	59.41	12.52	27.45
1.50	3.00	15.79	65.18	14.13	30.46
2.00	3.00	13.88	59.17	12.15	29.45
2.50	3.00	11.19	53.16	9.84	26.90
3.00	3.00	11.11	45.84	9.72	24.49

**Table 8:** Sample of the experimental array. Partial dependence on ***C_AS-2_***.

***C_AS-2_***, g/dm^3^	Grade of Crushed Stone			
	“KazRosResurs”		“Korneevka”	
	*W_A_KS_*, mN/m	*A_KS_*, %	*W_A_KA_*, mN/m	*A_KA_*, %
0.50	10.93	42.39	9.38	21.38
1.00	12.41	50.19	10.76	25.45
1.50	14.07	56.91	12.27	28.62
2.00	12.57	51.60	10.74	27.06
2.50	10.43	45.24	8.90	24.84
3.00	10.83	39.46	9.20	22.56

**Table 9:** Sample of the experimental array. Partial dependence on ***C_AG-4I_***.

***C_AG-4I_***, g/dm^3^	Grade of Crushed Stone			
	“KazRosResurs”		“Korneevka”	
	*W_A_KS_*, mN/m	*A_KS_*, %	*W_A_KA_*, mN/m	*A_KA_*, %
0.50	11.60	39.77	9.74	23.61
1.00	11.22	43.24	9.46	23.82
1.50	11.03	45.11	9.55	24.85
2.00	11.84	49.01	10.18	25.30
2.50	12.56	52.78	10.88	25.42
3.00	12.97	55.90	11.41	26.91

## Data Availability

The datasets generated and/or analyzed during the current study are available from the corresponding author upon reasonable request.
